# Helpful Ailments

**Published:** 1892-05

**Authors:** 


### HELPFUL AILMENTS.

In England gout is a very prevalent and painful disease. In this country it is less common. Our climate inclines us to excessive mental activity. As a result of this the brain appropriates nervous force at the expense of the digestive system, and so disposes us to dyspepsia ; but dyspepsia compels its victims in spite of themselves, to indulge somewhat sparingly in rich food, in the too free eating of which gout originates.

Of the two diseases, dyspepsia is to be preferred. It seldom interferes with the day's work, and, except in very obstinate cases, is almost certain to be relieved by proper diet and exercise.

Sick headache may often be counted in the class of helpful ailments, though it is a "bitter pill." There are two forms of it ; one has its primary source in the brain, the other in the stomach. In both cases there is commonly some hereditary tendency to the disease, but the exciting cause is overwork ; of the brain in one case ; of the stomach in the other.

The headaches necessitate occasional rest while the dread of them acts as a constant check upon the tendencies which might otherwise result in grave harm. Indeed, attention to diet, with a little letting down of the average cerebral activity, professional, business or domestic, will generally insure a comparative immunity from attack.

Acute rheumatism often gives rise to permanent heart trouble. Chronic rheumatism on the contrary, may be healthful in cases of heart disease.

For instance, enlargement of the heart, tends to increase until it reaches the dangerous limit. The patient's safety depends largely on his training himself to such habits as reduce strain on the heart, and rheumatic joints in the lower limbs are an admirable aid in this respect. The former rapid movements cease. A fatal running to meet the cars or the ferryboat is out of the question.

The rheumatism is an uncomfortable companion, no doubt, but it may help to a long life—a decade or more, perhaps beyond the three score and ten.

